# Chemovirotherapy of Lung Squamous Cell Carcinoma by Combining Oncolytic Adenovirus With Gemcitabine

**DOI:** 10.3389/fonc.2020.00229

**Published:** 2020-02-25

**Authors:** Xing Liu, Zhiguang Yang, Yiquan Li, Yilong Zhu, Wenjie Li, Shanzhi Li, Jing Wang, Yingli Cui, Chao Shang, Zirui Liu, Gaojie Song, Ce Li, Xiao Li, Guoguang Shao, Ningyi Jin

**Affiliations:** ^1^Department of Thoracic Surgery, The First Hospital of Jilin University, Changchun, China; ^2^Institute of Military Veterinary Medicine, Academy of Military Medical Science, Changchun, China; ^3^Academician Workstation of Jilin Province, Changchun University of Chinese Medicine, Changchun, China; ^4^Jiangsu Co-innovation Center for Prevention and Control of Important Animal Infectious Diseases and Zoonoses, Yangzhou, China; ^5^Department of Breast Surgery, The Second Hospital of Jilin University, Changchun, China; ^6^Department of Oncology Gynecology, The First Hospital of Jilin University, Changchun, China

**Keywords:** Apoptin, hTERT, recombinant adenovirus, gemcitabine, lung squamous cell carcinoma

## Abstract

Oncolytic virotherapy is emerging as an important agent in cancer treatment. In a previous study, we designed and constructed Ad-Apoptin-hTERTp-E1a (Ad-VT), a dual cancer-selective anti-tumor recombinant adenovirus. In this study, crystal violet staining and WST-1 assays showed that Ad-VT has a significant tumor killing effect in a time and dose dependent manner. The combination of Ad-VT (10 MOI) and gemcitabine (10 nM) significantly inhibited NCI-H226 cells, but did not increase the killing effect of gemcitabine on human normal bronchial epithelial cells BEAS-2B. Hoechst, JC-1 and Annexin V experiments demonstrated that the combination of Ad-VT and gemcitabine mainly inhibited NCI-H226 cell proliferation by inducing apoptosis (mitochondrial pathway). The combination also significantly inhibited the migration and invasion abilities of NCI-H226 cells. *In vivo*, Ad-VT in combination with low-dose gemcitabine could effectively inhibit tumor growth and prolong survival of mice. Ad-VT has the characteristics of tumor-selective replication and killing, *in vitro* and *in vivo*. The combined application of Ad-VT and gemcitabine has a synergistic effect, which can increase the anti-tumor effect and reduce the toxicity of chemotherapy drugs, indicating that Ad-VT has a potential clinical value in the treatment of lung squamous cell carcinoma.

## Introduction

Squamous cell lung cancer (SqCLC) is a distinct histologic subtype of NSCLC, accounting for 25–30% of cases (1). For SqCLC, the EGFR gene mutation rate is <4% and the rearrangement rate of the ALK gene is <3% (2–4). The main treatments of SqCLC rely on chemotherapy and radiotherapy. Chemotherapy of SqCLC employs gemcitabine combined with cisplatin, which is the currently preferred first-line treatment for a locally limited or advanced SqCLC according to the NCCN guidelines (5, 6). During chemotherapy, there are various degrees of side effects, such as nausea and vomiting, dry mouth, lack of appetite, hands and feet numbness, hair loss and anemia, which result in a general decline in patients' quality of life and in some cases forcing them to discontinue the treatment due to drug intolerance. Moreover, drug resistance is a major challenge in chemotherapy and therefore, the development of new therapeutic approaches for SqCLC has become an essential research topic in the field.

Oncolytic viruses (OVs) are emerging as important agents in cancer treatment as they offer the attractive therapeutic combination of tumor-selective cell lysis and by acting as potential *in situ* tumor vaccines (7–12). Early clinical trials (13–15) of OVs showed encouraging safety profiles, even at high doses, and with some promising responses, such as the evidence of intratumor viral replication and immune cells infiltration.

Apoptin was originally identified as the apoptosis inducing VP3 protein from chicken anemia virus (CAV), the first member of the Gyrovirus genus (16, 17). Apoptin is a small 14 kDa protein that is rich in proline, serine, threonine and basic amino acids. It has the ability to selectively kill various human tumors or transformed cells with little cytotoxic effects in normal cells (18–21).

Telomerase is an RNA-dependent DNA polymerase that elongates 5'-TTAGGG-3' telomeric DNA (22, 23). Most normal human somatic cells lack telomerase activity due to the tight transcriptional suppression of the human telomerase reverse transcriptase (hTERT) gene, a rate-limiting and catalytic component of telomerase enzyme. However, hTERT expression and telomerase activation are observed in up to 90% of human malignances, giving them an unlimited proliferation ability (24, 25). High hTERT expression is associated with advanced stages and unfavorable prognoses in different types of malignancies (26, 27).

In a previous study, we constructed a dual cancer-selective anti-tumor recombinant adenovirus. Ad-Apoptin-hTERTp-E1a (Ad-VT), that allows the adenovirus to selectively identify cancer cells, proliferate in large numbers, expresses the apoptin protein, and trigger tumor cell death (28). We showed that the modified adenovirus has a significant killing effect on several tumor cells (29–32). Another study evaluated the preclinical safety of Ad-VT and demonstrated that it has no obvious adverse effects on the central nervous, cardiovascular, respiratory or gastrointestinal systems (31).

As discussed above, chemotherapeutic drugs have certain limitations in treating SqCLC and recombinant adenoviruses can selectively recognize and kill tumors with few side effects. In this study, we decided to use the recombinant adenovirus Ad-VT in combination with gemcitabine to determine the optimal combinational concentration that allows *in vivo* and *in vitro* experimentations, with the expectation of achieving a reduced toxicity of gemcitabine and increased SqCLC treatment efficacy.

## Materials and Methods

### Cell Lines

The NCI-H226 human lung squamous carcinoma cell line, the BEAS-2B human normal bronchial epithelial cell line and the HEK-293 human embryonic kidney cell line were obtained from the Committee on Type Culture Collection of Chinese Academy of Sciences (Shanghai, China). The HEK-293 cells were cultured in 10% Dulbecco's modified Eagle's medium (DMEM; HyClone, USA). The NCI-H226 and BEAS-2B cells were cultured in 10% Roswell Park Memorial Institute (RPMI) 1640 medium (HyClone, USA). All media were supplemented with 10% fetal bovine serum (FBS; Hyclone, USA) and 1% Penicillin-Streptomycin (Sigma-Aldrich) at 37°C and in an atmosphere containing 5% CO_2_.

### Animals

The female Nude mice (3–4 weeks old with a weight of 16–22 g ± 0.25 g) were housed in light, comfortable temperature and humidity room (22 ± 2°C, 50 ± 5% relative humidity), given solid diet (Changchun billion Adams Laboratory Animal Technology Co., Ltd.) and sterilized tap water. All animals were obtained from the Experiment Animal Center of the Chinese Military Medical Academy and fasted before the experiments. The animal experimental protocols were approved by the Institutional Animal Care and Use Committee of the Chinese Academy of Military Medical Science, Changchun, China (10ZDGG007).

### Recombinant Adenoviruses

Recombinant adenoviruses Ad-Apoptin-hTERTp-E1a (Ad-VT), Ad-hTERTp-E1a (Ad-T), Ad-Apoptin (Ad-VP3), and Ad-Mock were constructed and preserved in our laboratory (Laboratory of molecular Virology and Immunology, Military Medical Science Academy of the PLA, Changchun, China) (28).

### Cell Inhibition Assays

The NCI-H226 and BEAS-2B cells were cultured in 96-well plates at a density of 5 × 10^3^ cells/well. Recombinant adenoviruses and gemcitabine (#S1714; Selleck Chemicals, Houston, TX, USA) were infected with the above cells at 24, 48, and 72 h 10 μL WST-1 solution (No. 11644807001; Cell proliferation assay reagent, Roche Applied Science, Mannheim, Germany) was mixed with 90 μL serum-free medium and added to each well for 90 min. Subsequently, the absorbance was measured at 450 nm with a 20 s shaking. The cell viability was calculated as follows: Cell inhibition ratio = 100% × [1 − OD (absorbance of treated wells)/OD (absorbance of control wells)].

The NCI-H226 and BEAS-2B cells were cultured in 12-well plates at a density of 2 × 10^5^ cells/well. For the crystal violet assay, after above treatment, the culture medium in each well was discarded and the plates were turned over on a filter paper for 1 min, and then 350 μL of 0.4% crystal violet solution (C0121; Beyotime technology, Shanghai, China) were added to each well for the staining at room temperature for 10 min. The staining solution was carefully sucked dry by turning the plates over on a filter paper.

### Analysis of Combination Synergy

The inhibition ratios of NCI-H226 and BEAS-2B cells were examined by the WST-1 assay as described above, and the combination index (CI) values were calculated from the CI equation algorithms (33) using CalcuSyn software analyses (Biosoft v2.0). CI = 1, < 1 and > 1 indicate additive, synergism and antagonism effect, respectively.

### Hoechst Assay

NCI-H226 cells at logarithmic growth phase were cultured at 2.5 × 10^5^ cells/well in 6-well cell culture plates, which were pre-inserted with cover glass slides, and cultured at 37°C in 5% CO_2_ incubator for 24 h. After treatment, the medium was discarded and washed 3 times with phosphate-buffered saline (PBS). Hoechst (H21486; Life technologies, Invitrogen, USA) dye was diluted 1:1000 and each well was added with 1 mL Hoechst staining fluid. After 8 min of incubation at 37°C with 5% CO_2_, the cover slips were washed 2 times with PBS and then observed and photographed under a fluorescence microscope (BX-60; Olympus, Tokyo, Japan). Tests were performed in triplicate and at least 300 cells were scored for each sample to determine the nuclear changes.

### Measurement of Apoptosis by Flow Cytometry

Logarithmic growth phase NCI-H226 cells were cultured at 2.5 × 10^5^ cells/well in 6-well cell culture plates, and at 37°C with 5% CO_2_ for 24 h. After treatment, the cells were harvested, resuspended in binding buffer and stained with fluorescein isothiocyanate (FITC) labeled Annexin V (Annexin V-FITC Apoptosis Detection kit; 556419, BD Pharmingen, USA) according to the manufacturer's protocol. To exclude late apoptotic and necrotic cells, propidium iodide (PI; 2.5 μg/mL) (Annexin V-FITC Apoptosis Detection kit; 556463, BD Pharmingen, USA) was added to the FITC-Annexin V-stained samples and incubated at room temperature for 30 min. The samples were then examined by flow cytometry (FACS Calibur; BD Biosciences, Franklin Lakes, NJ, USA) for apoptosis analysis (Cell Quest Pro 5.2.1; BD Biosciences).

### Cell Cycle Analysis

NCI-H226 cells at logarithmic growth phase were cultured at 2.5 × 10^5^ cells/well in 6-well cell culture plates, and at 37°C with 5% CO_2_ for 24 h. The cells were harvested at 72 h and centrifuged at 175 × g for 5 min at room temperature. Discarded the supernatant and washed the pellet 1 time with 1 × PBS. The cells were harvested and fixed overnight in 75% ethanol at 4°C. The next day, they were washed with 1 × PBS and centrifuged at 175 × g for 10 min at room temperature and the supernatant discarded. The cells were then mixed with 0.09% NaN_3_ Stain Buffer (2% FBS), washed and centrifuged at 175 × g for 10 min at room temperature, and the supernatant discarded. Finally, 500 μL PI/RNase (PI/RNase Staining Buffer; 550825, BD Pharmingen, USA) staining solution was used to dye the cells for 30 min at room temperature. Flow cytometry was performed using a FACS Calibur instrument and Cell Quest Pro 5.2.1 software.

### JC-1 Assays

NCI-H226 cells (2.5 × 10^5^ cells/well in 6-well cell culture plates which were pre-inserted with cover glass slides) were cultured at 37°C and in 5% CO_2_ for 24 h. Treated cells were harvested at 72 h, JC-1 (T3168; Invitrogen, Life technologies, USA) fuel was diluted 1:1000, and 1mL JC-1 diluent was added to each well and incubated for 15 min in the dark. The cover glass slides were washed 3 times with 1×PBS and then the cells were observed and photographed using fluorescence microscopy.

NCI-H226 cells (5 × 10^3^ cells/well in 96-well plates) were cultured at 37°C and in 5% CO_2_ for 24 h. Treated cells were harvested at 72 h. JC-1 fuel was diluted 1:1000 and 100 μL of JC-1 solution was added to each well, incubated at 37°C in 5% CO_2_ for 15 min in the dark, and then washed 3 times with PBS. Subsequently, the absorbance at 485–530 nm and 530–590 nm were measured using a microplate reader. Red/Green ratio = 100%× OD (530–590 nm)/OD (485–530 nm).

### Transwell Migration and Biocoat Invasion Assays

The Transwell (REF3422; Corning Incorporated, COSTA, USA) chamber was placed in a culture plate, and the treated cells (2.0 × 10^5^ cells/well in 24-well cell culture plates) were harvested and resuspended in 200 μL serum-free 1640 medium. The cell suspension was added to the upper chamber and 600 μL 10% 1640 medium was added to the lower chamber. The cells in the upper layer of the membrane were removed with cotton swabs after 24 h of culture at 37°C and 5% CO_2_. After methanol fixation, crystal violet staining and microscope observation, photographs were taken. The cells which migrated through the membrane were counted.

The BioCoat (REF354480; Corning, USA) invasion chamber was removed from the −20°C refrigerator and warmed to room temperature. 500 μL of preheated (37°C) serum-free medium was added to the cell chamber of the culture plate. This step was followed by 2 h in a CO_2_ free environment at 37°C. NCI-H226 cells (2.0 × 10^5^ cells/well in 24-well cell culture plates) were harvested, resuspended in 200 μL serum-free medium and seeded in the upper chamber after treatment. 500 μL 10% 1640 medium was added to the lower chamber and incubated for 24 h at 37°C with 5% CO_2_. The cells which migrated through the membrane were counted under a microscope after fixation with carbinol and staining with crystal violet.

### Cell Scratch Assay

NCI-H226 cells were cultured at 1 × 10^6^ cells/well in 6-well cell culture plates, at 37°C and 5% CO_2_ for 24 h. A sterile micropipette tip was used to make a scratch in the cell layer from one end of the 6-well cell culture plate to the other. The cells were washed 3 times with PBS and images were captured under an inverted microscope. The 6-well cell culture plates were photographed under an inverted microscope after 72 h post-treatments and the scratches width were measured. Cell migration was calculated according to the following formula: Cell migration ratio = 100% × (0 h scratch width – 72 h scratch width)/0 h scratch width.

### Tumor Xenograft Experiments

NCI-H226 cells (1 × 10^7^/100 μL) were injected into the right hind leg of nude mice. The xenograft models were infected with Ad-VT (1 × 10^9^ plaque forming unit, PFU; biweekly) via intratumor injection and gemcitabine (30, 60, and 120 mg/Kg; weekly) via intraperitoneal injection. The length and width of the xenograft tumors were weekly measured using a vernier caliper. Body weight was also measured. The tumor volume was calculated using the following formula: Tumor volume (mm^3^) = Longest diameter × shortest diameter^2^/2. The percent of tumor inhibition was calculated using the formula: 100% × (1 − treatment group tumor volume/control tumor volume). The survival of the nude mice was daily recorded.

For the inhibitory effects of the combination of Ad-VT with low-dose gemcitabine on the xenografts, the mice were given gemcitabine (10, 20 mg/Kg) weekly and Ad-Mock (1 × 10^9^ PFU) and Ad-VT (1 × 10^9^ PFU) biweekly. The methods of administration, tumor measurement and survival analysis of mice were the same as above.

### Histology

Mice liver and kidney tissues were extracted, immersed in 12% formalin and fixed for more than 24 h. The fixed tissues were under water flows overnight and then dehydrated by an automatic dehydrator. The embedding machine was used for paraffin embedding. One of the slides containing tissue sections was deparaffinized, rehydrated using a dry oven at 65°C for 30 min, dipped in two changes of xylene for 10 min in a series of decreasing concentrations of alcohol and finally in tap water. The slides were soaked in hematoxylin for 8 min and rinsed under running water. The slides were then soaked in eosin for 2 min and rinsed under running water. After dehydration with alcohol and xylene, the slices that were dehydrated and transparent, were sealed with neutral resin and air-dried for 1 day.

### Statistical Analysis

The data were presented as mean ± standard error of the mean (SEM). GraphPad Prism 5.0 software was used to perform statistical analyses of unpaired two-tailed Student's *t* tests or analysis of variance (ANOVA). *P* < 0.05 was considered to be statistically significant. The Log-Rank test was used to compare survival between the groups. ^*^p < 0.05, ^**^p < 0.01, ^***^p < 0.001, and ^****^p < 0.0001.

## Results

### Ad-VT Effectively Kills NCI-H226 Cells in a Time and Dose Effect-Dependent Manner

**Figure 1:**
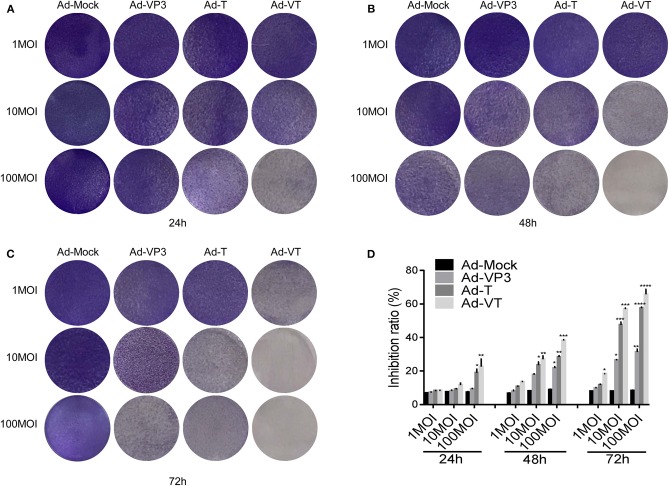
Ad-VT effectively kills NCI-H226 cells in a time and dose effect-dependent manner. **(A–C)** NCI-H226 cells in 12-well plates were infected with Ad-VT, Ad-T, Ad-VP3, and Ad-Mock at 1 MOI, 10 MOI, and 100 MOI, respectively, and then stained with 0.4% crystal violet at 24 **(A)**, 48 **(B)** and 72 h **(C)**. **(D)** NCI-H226 cell inhibition ratio was determined using the WST-1 assay after Ad-VT, Ad-T, Ad-VP3, and Ad-Mock treatment at various concentrations (1MOI, 10MOI, and 100 MOI) for 24, 48, 72 h. Data were representative of three independent experiments (n = 3). One-way analysis of variance (ANOVA) was used. *p < 0.05, **p < 0.01, ***p < 0.001, ****p < 0.0001. Recombinant adenoviruses: Ad-Apoptin-hTERTp-E1a (Ad-VT), Ad-hTERTp-E1a (Ad-T), Ad-Apoptin (Ad-VP3) and Ad-Mock.

First, we tested whether the Ad-VT could inhibit NCI-H226 by crystal violet assay. We found that Ad-VP3, Ad-T, and Ad-VT could significantly inhibit the proliferation of NCI-H226 cells when compared with Ad-Mock and in a time and dose dependent manner. The strongest inhibitory effect was mediated by Ad-VT, followed by Ad-T and Ad-VP3 (Figures 1A–C).

Subsequently, the WST-1 assay was used to further detect the cell inhibition rate. At 1 MOI, the inhibition ratio of Ad-VT was 18.34% at 72 h (*p* < 0.05). At 10 MOI, Ad-VP3, Ad-T and Ad-VT increased the inhibition ratio of NCI-H226 proliferation with time. The inhibition rate of Ad-VP3 on NCI-H226 was 27.16% at 72 h. From 48 h to 72 h, the inhibition of Ad-T on NCI-H226 cells was between 22.16and 48.37% (*p* < 0.05), while the inhibition of NCI-H226 proliferation by Ad-VT fluctuated between 22.66and 56.49% (p < 0.01). At 100 MOI, the inhibitory effect of Ad-VP3, Ad-T, and Ad-VT on NCI-H226 cells significantly increased with time (*P* < 0.05). In the process of infection, the inhibition rate of Ad-VT, Ad-T, and Ad-VP3 on cells, reached the maximum at 72 h with 69.69, 57.91, and 30.16%, respectively (*p* < 0.01) (Figure 1D).

The above results suggested that recombinant adenovirus Ad-VP3, Ad-T, and Ad-VT showed different degrees of inhibition on NCI-H226 cells and in a time and dose dependence fashion. The strongest inhibitory effect was mediated by Ad-VT, followed by Ad-T and Ad-VP3.

#### Ad-VT Increases the Anti-tumor Effect and Relatively Reduces the Toxicity of Gemcitabine

**Figure 2:**
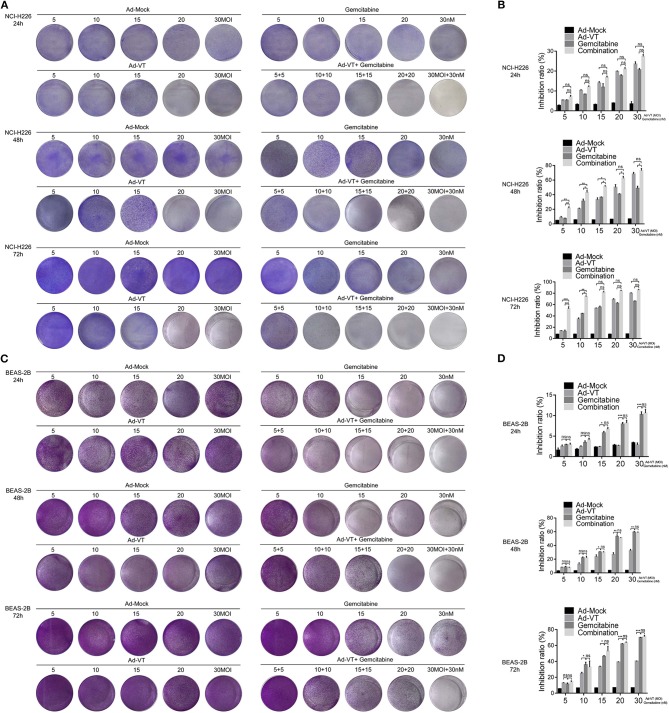
Ad-VT increases the anti-tumor effect and relatively reduces the toxicity of gemcitabine**. (A)** The anti-tumor effect of Ad-VT, gemcitabine and combination group on NCI-H226 at 24, 48, and 72 h by crystal violet assay. **(B)** WST-1 assay measured the inhibition of Ad-VT, gemcitabine and combination group on NCI-H226 at 24, 48, and 72 h. **(C)** The toxicity effect of Ad-VT, gemcitabine and combination group on BEAS-2B at 24, 48, and 72 h by crystal violet assay. **(D)** WST-1 assay measured the inhibition of Ad-VT, gemcitabine and combination group on BEAS-2B at 24, 48, and 72 h. Data were representative of three independent experiments (*n* = 3). The unpaired Student's *t* test was used. *p < 0.05, **p < 0.01, ***p < 0.001.

NCI-H226 cells were treated in a 1:1 proportion with gemcitabine at concentrations of 5, 10, 15, 20, and 30 nM and with Ad-VT at 5, 10, 15, 20, and 30 MOI. The results of the crystal violet experiment showed that the staining degree of cells in Ad-VT, gemcitabine and combination groups was significantly reduced and with a decreasing trend at 24, 48, and 72 h post-treatments. The combination group showed the strongest inhibitory effect on NCI-H226, especially at 72 h post-treatment (Figure 2A).

The inhibitory effect of gemcitabine and Ad-VT on NCI-H226 was detected by the WST-1 assay. 24 h post-treatments, there were no significant differences between three groups. However, at 48 h, the results showed that the inhibitory effects on NCI-H226 cells by Ad-VT, gemcitabine and the combination group increased in a dose dependent fashion. The inhibition rate of gemcitabine alone treated group was 7.47, 30.56, 35.86, 40.86, and 45.47%, respectively. The inhibition rate of Ad-VT group was 7.57, 21.59, 35.45, 46.51, and 65.51%, respectively. The inhibition rate of the combination group was 23.46, 45.66, 50.11, 60.77, and 75.87%, respectively. The inhibition rate in the combination group was significantly higher than that of the gemcitabine alone treated group (*p* < 0.05). The inhibition rates in the 5 nM + 5 MOI, 10 nM + 10 MOI, and the 15 nM + 15 MOI combination groups were significantly higher than that of the 5 MOI, 10 MOI, and 15 MOI Ad-VT treated group (*p* < 0.05). At 72 h, the inhibition rate of gemcitabine alone treated group was 11.31, 44.66, 57.11, 63.39, and 65.41%, respectively. The inhibition rates of Ad-VT group were 13.31, 36.27, 54.06, 70.38, and 80.52%, respectively. The inhibition rates of the combination group were 50.09, 75.38, 82.34, 84.68, and 86.13%, respectively. The inhibition rates of the combination group were only significantly different from the gemcitabine and Ad-VT single agent groups with treatments of 5 nM + 5 MOI, and 10 nM + 10 MOI (*p* < 0.05) (Figure 2B).

We further tested the toxic effects of Ad-VT, gemcitabine and the combination group on the normal bronchial epithelial cells BEAS-2B by the crystal violet assay. The results showed that after staining with 0.4% crystal violet, there were no significant changes between the Ad-VT group (from 5 MOI to 30 MOI) at 24, 48, and 72 h. The staining intensity of the gemcitabine group showed a decreasing trend from 5 to 30 nM at 24, 48, 72 h. The staining intensity of the combination group was similar to that of the gemcitabine group, which showed a decreasing trend (Figure 2C).

Moreover, we used WST-1 assay to detect the inhibitory effect of gemcitabine and Ad-VT and combination on BEAS-2B cells.

At 24, 48 h post-treatments, the results showed that the inhibition rate of gemcitabine was significantly higher than that of Ad-VT at the concentrations of 15, 20, and 30 nM (*p* < 0.05). There was no statistically significant difference in the inhibition rate between the combination group and the gemcitabine alone treated group. At 72 h post-treatment, there were differences between the gemcitabine group and the Ad-VT group at the concentrations of 10, 15, 20, and 30 (*p* < 0.05), indicating that the toxicity effect of Ad-VT on normal lung cells was significantly lower than that of gemcitabine. Moreover, there was no difference between the combination group (15.67, 25.76, 59.5, 62.34, and 69.59%) and the gemcitabine group (11.31, 34.66, 47.11, 60.97, and 69.74%) (Figure 2D).

The above results indicated that Ad-VT has strong inhibitory effect on tumor cells and the addition of Ad-VT does not increase the inhibition rate of gemcitabine on normal cells, but the combination of Ad-VT and gemcitabine has a strong inhibition effect on cancer cells.

### Ad-VT Synergizes With Gemcitabine on NCI-H226 Cells

**Figure 3:**
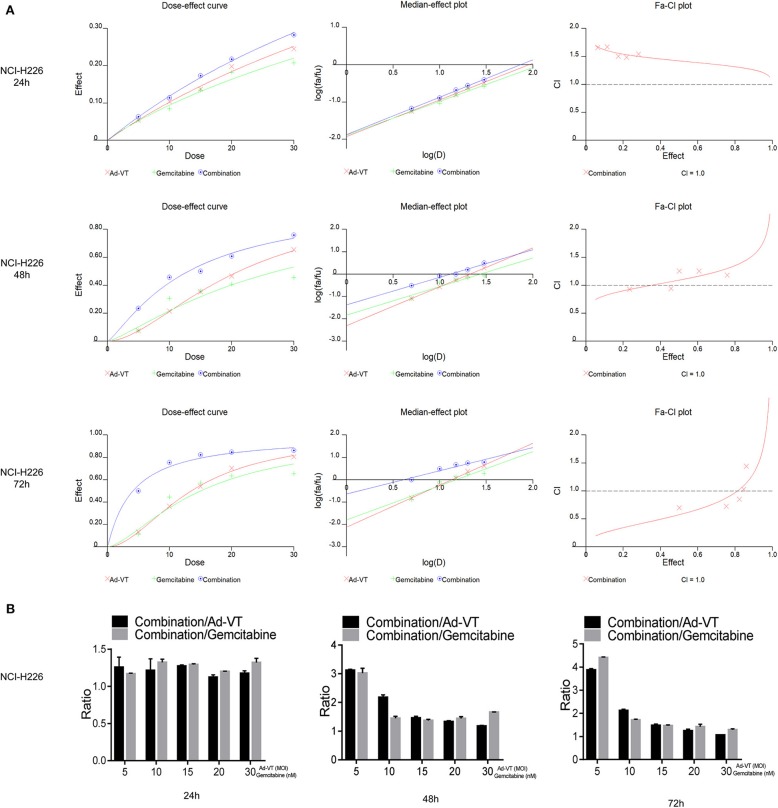
Ad-VT synergizes with gemcitabine on NCI-H226 cells. **(A)** The CalcuSyn software calculated CI of the combination group at 24, 48, and 72 h. **(B)** Ratios of combination/Ad-VT, combination/gemcitabine at 24, 48, and 72 h.

By introducing the above data into the CalcuSyn software, it can be concluded that at 48 h post-treatments, the CI of the combination groups of 5 nM + 5 MOI, 10 nM + 10 MOI, were <1. At 72 h post-treatments, the CI of the combination groups of 5 nM + 5 MOI, 10 nM + 10 MOI, and 15 nM + 15 MOI, were <1, indicating a synergistic inhibitory effect (Figure 3A).

By calculating the ratios of the inhibition rate of the combination/Ad-VT and the combination/gemcitabine, we found that at 48, 72 h, these ratios decreased with increasing doses, indicating that the effect of the combination therapy, at low dose, was much higher than that of the single-dose group (Figure 3B).

Above all, the combination of gemcitabine and Ad-VT 5 nM + 5 MOI, 10 nM + 10 MOI, and 15 nM + 15 MOI had synergistic inhibitory effects (CI < 1) at 72 h post-treatments, with inhibition rates of 50.09, 75.38, and 82.34%, respectively. The combinations of 5 nM + 5 MOI, 10 nM + 10 MOI were significantly different from the single agent group (*p* < 0.05). For normal cells, the inhibition rate of the combination of the two, showed no statistical difference when compared with the gemcitabine group, indicating that the addition of Ad-VT does not increase the inhibition rate of gemcitabine on normal cells. According to the above results, the combination of 10 nM + 10 MOI can reduce toxicity and increase efficiency.

### Combination of Ad-VT and Gemcitabine Induces Selective Apoptosis of NCI-H226 Cells Through Mitochondrial Pathway and Changes Their Cell Cycle

**Figure 4:**
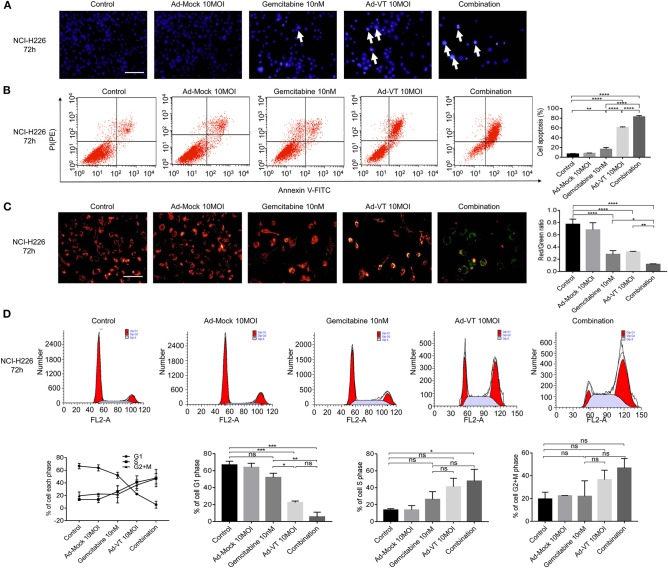
Combination of Ad-VT and gemcitabine induces selective apoptosis of NCI-H226 cells, through mitochondrial pathway, and changes their cell cycle. **(A)** Hoechst test showing that the nuclei of NCI-H226 cells infected with Ad-VT 10 MOI, gemcitabine 10 nM and combination 10 MOI + 10 nM group presented different degrees of bright blue hyperchromatism or fragmentation. The number of cells showed a decreasing trend. **(B)** Annexin V results showing the apoptosis rates of gemcitabine 10 nM, Ad-VT 10 MOI and the combination 10 nM + 10 MOI. **(C)** JC-1 experiment showing the mitochondrial membrane potential and the ratio of red/green of the different treatment group. The apoptotic cells gradually increased and the JC-1 gradually changed from the initial red aggregate to the green monomer. **(D)** The change of the cell cycle when treat with Ad-VT 10 MOI, gemcitabine 10 nM and combination 10 MOI + 10 nM group. The scale bar equals 100 μm. Data were representative of three independent experiments (*n* = 3). The unpaired Student's *t* test was used. *p < 0.05, **p < 0.01, ***p < 0.001, ****p < 0.0001.

Hoechst results showed that the nuclei of NCI-H226 cells that treated with Ad-VT 10 MOI, gemcitabine 10 nM and a combination of 10 MOI + 10 nM group presented different degrees of bright blue hyperchromatism or fragmentation and the number of cells showed a decreasing trend while the nuclei of the Ad-Mock group showed a uniform blue fluorescence (Figure 4A). Hoechst results suggested that nuclear changes occurred in both the single and combined treatment groups, suggesting apoptosis after treatment, and the combined treatment group had the most obvious effect.

Another experiment, which confirmed the evidence of apoptosis induction, was the Annexin V assay. Ad-VT 10 MOI, gemcitabine 10 nM and the combination 10 MOI + 10 nM groups can induce apoptosis, but the degree of apoptosis was different. The results showed that the apoptosis rates of gemcitabine 10 nM, Ad-VT 10 MOI and the combination 10 nM + 10 MOI groups were 12.86, 60.68, and 82.3%, respectively; and the apoptosis rate was significantly different compared with the control group (*p* < 0.01). The comparison between the gemcitabine 10 nM, the Ad-VT 10 MOI and the combination 10 nM + 10 MOI groups showed that the apoptosis rate of the combined group was significantly higher than that of the single group (*p* < 0.0001), and the apoptosis rate of the Ad-VT group was significantly different from that of the gemcitabine group (*p* < 0.0001) (Figure 4B). Flow cytometry results of Annexin V showed consistence with that of the WST-1 experiment, further confirming that gemcitabine, Ad-VT and the combination group inhibited cells by inducing apoptosis.

The changes of the mitochondrial membrane potential (MMP) of NCI-H226 cells treated with Ad-VT, gemcitabine and combination groups were detected by JC-1 experiment. The results showed, that compared with the control group and the Ad-Mock group, MMP depolarized. The apoptotic cells gradually increased and the JC-1 gradually changed from the initial red aggregate to the green monomer (Figure 4C). The results of the microplate assay showed that the red-green light ratios of gemcitabine, Ad-VT and the combination groups were 0.24, 0.31, and 0.11, which were significantly lower than that of the control and Ad-Mock groups (*p* < 0.0001). The red-green light ratio of the combination group was significantly lower than that of the single agent group (*p* < 0.05) (Figure 4C). These results suggest that the combined treatment group can induce mitochondrial depolarization.

The results of cell cycle showed that in the G1 phase, compared with the control group, Ad-VT group and the combination group showed a significantly lower trend (*p* < 0.001), while there was no significant difference between the gemcitabine group and the control group. The percentage of G1 cells in the Ad-VT group and the combination group was significantly lower than that in the gemcitabine group (*p* < 0.05), and there was no significant difference between the Ad-VT group and the combination group, indicating that Ad-VT could significantly reduce the percentage of G1 cells. The percentage of S phase cells in the combined group was significantly higher than that in the control group (*p* < 0.05) (Figure 4D). The above results showed that Ad-VT can affect the cell cycle at G1-S transition phase. We suggest that the increased DNA synthesis during this phase may be a self-protective mechanism of tumor cells.

Above all, the combination group had the strongest ability to induce apoptosis through MMP changes, with the highest number of apoptotic cells and the most significant decrease in the red/green fluorescence ratio.

### Combination of Ad-VT and Gemcitabine Inhibits the Migration and Invasion Abilities of NCI-H226 Cells

**Figure 5:**
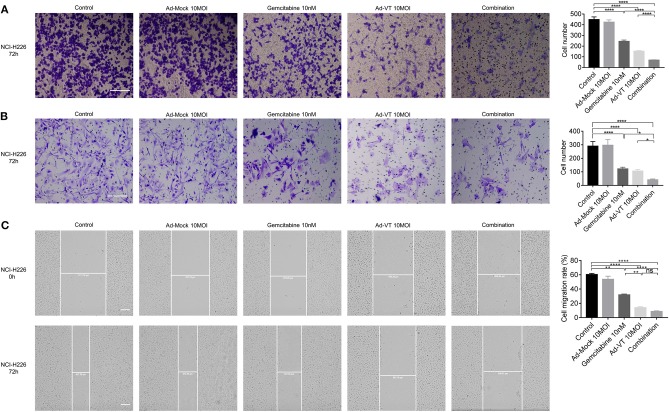
Combination of Ad-VT and gemcitabine inhibits the migration and invasion abilities of NCI-H226 cells. **(A)** The Transwell assay detected the migration ability of NCI-H226 cells after Ad-VT 10 MOI, gemcitabine 10 nM and combination 10 MOI + 10 nM group at 72 h. **(B)** The BioCoat assay examined the invasion ability of NCI-H226 when treated with Ad-VT 10 MOI, gemcitabine 10 nM and combination 10 MOI + 10 nM group at 72 h. **(C)** The scratch assay tested the migration ability of NCI-H226 when treated with Ad-VT 10 MOI, gemcitabine10 nM and combination 10 MOI + 10 nM group at 72 h. The scale bar equals 200 μm. The cell migration rate was calculated according to the following formula: Cell migration ratio = 100% × (0 h scratch width-72 h scratch width)/0 h scratch width. Data were representative of three independent experiments (*n* = 3). The unpaired Student's *t* test was used. *p < 0.05, **p < 0.01, ****p < 0.0001.

The Transwell assay showed that Ad-VT 10 MOI, gemcitabine 10 nM and the combination 10 MOI + 10 nM groups could inhibit the migration ability of NCI-H226 cells (*p* < 0.0001); while, Ad-Mock could not. The inhibition of migration ability was strongest in the following order: Combination group > Single agent group (*p* < 0.0001) (Figure 5A).

The results of BioCoat assay showed that Ad-VT 10 MOI, gemcitabine 10 nM and the combination 10 MOI + 10 nM groups could inhibit the migration ability of NCI-H226 cells (*p* < 0.0001). The inhibition of invasion ability was the strongest in the following order: Combination group > Single agent group (*p* < 0.05) (Figure 5B).

The results of the scratch test showed that the cell migration ability of each treatment group was significantly lower than that of the control group (*p* < 0.01). The cell migration ability of the combination group was significantly lower than that of the gemcitabine group (*p* < 0.0001), but there was no significant difference with the Ad-VT group. In the treatment group alone, the inhibition effect of Ad-VT on cell migration was significantly higher than that in the gemcitabine group (*p* < 0.01) (Figure 5C).

### Low-Dose Gemcitabine Combined With Ad-VT Can Reduce Toxicity and Increase Efficiency

**Figure 6:**
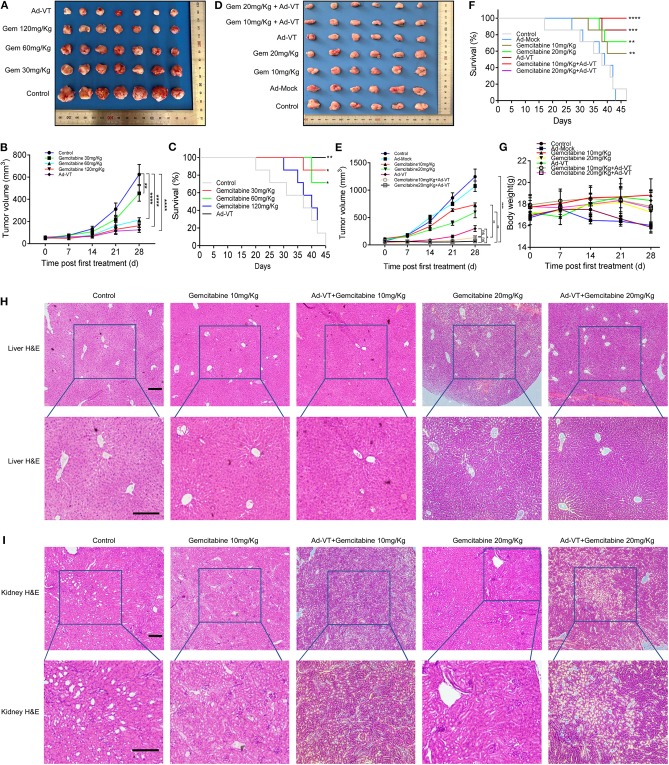
Low-dose gemcitabine combined with Ad-VT can reduce toxicity and increase efficiency. **(A,B)** Tumors from the nude mice treated with Ad-VT and gemcitabine, at different concentrations were excised, photographed and measured. **(C)** The survival rate of mice in the 30 mg/kg, 60 mg/kg and 120 mg/kg gemcitabine treatment groups and Ad-VT (1 × 10^9^ PFU) group. **(D,E)** The photos and measurement of subcutaneous tumors of gemcitabine 10 mg/kg, gemcitabine 20 mg/kg, Ad-VT (1 × 10^9^ PFU), gemcitabine 10 mg/kg + Ad-VT (1 × 10^9^ PFU), gemcitabine 20 mg/kg + Ad-VT (1 × 10^9^ PFU) group. **(F)** The survival rate of mice in the gemcitabine 10 mg/kg, gemcitabine 20 mg/kg, Ad-VT (1 × 10^9^ PFU), gemcitabine 10 mg/kg + Ad-VT (1 × 10^9^ PFU) and gemcitabine 20 mg/kg +Ad-VT (1 × 10^9^ PFU) group. (**G)** The body weight of mice in different treatment groups. **(H)** Liver H&E staining showed that the hepatocytes of all groups were radially arranged around the central vein. There was no significant difference between each group. **(I)** Kidney H&E staining, normal glomerular and renal tubules were observed in the control group. Kidney histology in the other treatment groups were similar to those in the control group. The scale bar equals 100 μm. Two-way analysis of variance (ANOVA) was used. The Log-Rank test was used to compare survival between the groups. *p < 0.05, **p < 0.01, ***p < 0.001, ****p < 0.0001.

First, we examined the inhibitory effects of Ad-VT and gemcitabine at different concentrations on the mice xenografts. Tumors' size was continuously measured for 4 weeks after subcutaneous tumor formation. The tumor volumes measured included the tumors from the gemcitabine 30, 60, 120 mg/kg, and Ad-VT (1 × 10^9^ PFU) treatment groups and the values were 453.45, 213.90, 162.12, and 125.30 mm^3^, respectively, and which were statistically different from the control group (624.09 mm^3^) (*p* < 0.01). The inhibitory rate was 27.34, 65.72, 74.02, and 79.92%, respectively (Figures 6A,B). In addition, the mice survival rates in the 30 mg/kg gemcitabine treated and 60 mg/kg gemcitabine treated groups were 85.71% and 71.43%, which were statistically significant when compared to the control group (*p* < 0.05). The mice survival rates in the 120 mg/kg gemcitabine treatment group was 14.29%. The Ad-VT (1 × 10^9^ PFU) group had the highest survival rate of 100% (*p* < 0.01) (Figure 6C). We found that with the increase of the gemcitabine dose, the survival rate of mice gradually decreased, with mice in the high-dose group fast dying.

Next, we examined the inhibitory effects of low-dose gemcitabine combined with Ad-VT on mice xenografts. Tumors' size was measured continuously for 4 weeks after subcutaneous tumor formation. The tumor volumes measured included the tumors from the gemcitabine 10, 20 mg/kg, Ad-VT (1 × 10^9^ PFU), gemcitabine 10 mg/kg + Ad-VT (1 × 10^9^ PFU) and gemcitabine 20 mg/kg + Ad-VT (1 × 10^9^ PFU) treated groups and the obtained values were 735.94, 597.40, 298.56, 135.63, and 67.63 mm^3^, respectively, which were statistically different from the control group (1245.93 mm^3^) (*p* < 0.0001). The inhibitory rate was 40.93, 52.05, 76.04, 89.11, and 94.57%. Tumor volumes of the combination group gemcitabine 20 mg/kg + Ad-VT (1 × 10^9^ PFU) were statistically different from the single agent group (gemcitabine 20mg/Kg, Ad-VT 1 × 10^9^ PFU) (*p* < 0.05). There were no differences between the two combination groups. For the gemcitabine 10 mg/kg + Ad-VT (1 × 10^9^ PFU) group, the tumor volumes were statistically lower than that of the single agent gemcitabine 10 mg/kg group (*p* < 0.01); However, there were no differences between the gemcitabine 10 mg/kg + Ad-VT (1 × 10^9^ PFU) and Ad-VT (1 × 10^9^ PFU) groups (Figures 6D,E).

For survival analysis, the mice survival rates in the 10 mg/kg and 20 mg/kg gemcitabine treated group were 57.14% and 71.43%, which was significant from that of the control group (*p* < 0.01). In the combination group, the mice survival rates in the gemcitabine 10 mg/kg + Ad-VT and gemcitabine 20 mg/kg + Ad-VT treatment groups had the highest survival rate of 100% (*p* < 0.0001) (Figure 6F).

Similarly, mice weight in the control group and Ad-Mock, significantly decreased over time; while, mice in the low-dose chemotherapy group and the combination group showed no significant fluctuation (Figure 6G).

The hepatocytes of the control group were radially arranged around the central vein. There was no significant difference in the livers of mice in the gemcitabine 10 mg/kg, Ad-VT + gemcitabine 10 mg/Kg, gemcitabine 20 mg/kg, and Ad-VT + gemcitabine 20 mg/Kg groups when compared with the control group (Figure 6H). For mice kidney H&E staining, normal glomerular and renal tubules were observed in the control group. Kidney histology in the other treated groups were similar to those in the control group (Figure 6I).

For the H&E staining, the combination group of Ad-VT with low dose gemcitabine showed no obvious histological changes in liver and kidney cells, which indicated that the combination group of Ad-VT, with low dose gemcitabine, had no obvious adverse effect on liver and kidney.

## Discussion

We observed that Ad-VT has the characteristics of tumor-selective replication and killing, *in vitro* and *in vivo*. The combined application of Ad-VT and gemcitabine has a synergistic effect, which can increase the anti-tumor effect and reduce the toxicity of chemotherapy drugs.

Gemcitabine is a new derivative of cytosine nucleoside and like cytarabine, it is activated by the deoxycytosine kinase and metabolized by the cytosine nucleoside deaminase (34, 35). In the field of anti-tumor therapy, more interest and fundings are being devoted to gene therapy. OVs are therapeutic approach to cancer treatment that utilize native or genetically modified viruses, that selectively replicate within tumor cells and kill cancer cells, or indirectly by immune mediated clearance of cancer cells and tumor vasculature selecting. The first reported genetically engineered OV was Talimogene Laherparepvec (T-VEC; Imlygic™), which has been approved in the US Food and Drug Administration (FDA) for metastatic melanoma as a single agent. In patients with unresectable metastatic melanoma, T-VEC demonstrated a superior durable response rate (continuous complete response or partial response lasting ≥ 6 months) over subcutaneous GM-CSF (16.3% vs. 2.1%; *p* < 0.001). Responses were seen in both injected and uninjected lesions, including visceral lesions, suggesting a systemic antitumor response (36, 37).

In a previous study, we constructed a dual cancer-selective anti-tumor recombinant adenovirus, designating it as Ad-VT (28). Briefly, transgene cassettes containing the hTERT core promoter and the Apoptin-driving cytomegalovirus promoter were subcloned into the adenoviral genome via a shuttle vector. The infectious adenovirus designated as Ad-VT was packaged in HEK-293 cells. This recombinant adenovirus possesses the ability to induce both tumor-selective growth inhibition and tumor-selective replication. Previous studies have shown that Ad-VT could inhibit the growth and migration of several malignant tumors. For the melanoma A375 and B16 cell lines, the infection with the adenoviruses expressing apoptin were observed predominantly in a late apoptotic stage. Also, Ad-VT conferred significant survival benefits *in vivo* (29–32). The mice lungs infected with Ad-VT had minimal metastatic nodules; whereas, the lungs from control or treated groups had severe metastasis. Taken together, the systemic delivery of Ad-VT significantly reduced tumor burdens and provided survival benefits in a lung metastatic cancer model. Previous research investigated preclinical safety profiles of Ad-VT in animal models, which showed that Ad-VT had no obvious adverse effects on BALB/C mouse behavior, muscle cooperation, sedative effect, digestive system or nervous systems, or on beagle cardiovascular and respiratory systems. (31).

At present, the combined application of chemotherapy drugs with oncolytic viruses, has attracted wide attention. It was reported that dl922-947 (an oncolytic adenovirus with a 24-bp deletion in E1A CR2) and low-dose paclitaxel induce aberrant, multipolar mitoses, mitotic slippage and multinucleation, triggering an apoptotic cell death (38).

In this study, we decided to use the recombinant adenovirus Ad-VT in combination with gemcitabine to determine the optimal combinational concentration that allows *in vivo* and *in vitro* experimentations, with the expectation of achieving a reduced toxicity of chemotherapy drugs and increased SqCLC treatment efficacy. We observed that although Ad-VP3 does not have the ability to self-replicate in tumor cells, the apoptin protein expression by Ad-VP3 plays an important role in tumor suppression. While Ad-T and Ad-VT carry hTERT promoters and can replicate in tumor cells, the inhibition rates of the two are higher than that of Ad-VP3. Ad-VT can both replicate in tumor cells and express the apoptin protein and is most effective in killing tumor cells in a time and dose effect-dependent manner. Theoretically, Ad-VT has a killing effect on BEAS-2B cells, which may also increase with time and dose. The inhibition effect of gemcitabine on BEAS-2B cells increased with time and dose. In general, Ad-VT has a better killing effect on lung cancer cells and a lower killing effect on normal cells; while, gemcitabine has a greater killing effect on normal cells. The inhibitory effect of the combination on NCI-H226 increased with time and dose. It indicates that the addition of Ad-VT does not increase the inhibition rate of gemcitabine on normal cells. The combination of Ad-VT and gemcitabine can mainly enhance the inhibition ability, by inducing apoptosis (Mitochondrial pathway) and inhibiting the growth, migration and invasion abilities of tumor cells.

It has been reported that the maximum tolerated dose of gemcitabine in mice is 120 mg/Kg (39). In this study, after the subcutaneous tumor model in nude mice was successfully generated, different concentrations of gemcitabine (30, 60. and 120 mg/Kg) and 1 × 10^9^ PFU of Ad-VT were given. We found that the high-dose gemcitabine group had high tumor inhibition rate, fast weight loss, high mortality and low survival rate. Therefore, when compared to the results of *in vivo* experiments, we reduced the dose of chemotherapeutic drugs and combined them with Ad-VT treatment to observe whether low-dose gemcitabine combined with Ad-VT could also reduce toxicity and increase efficiency. We found that the combination of low-dose gemcitabine and Ad-VT achieved similar or better inhibitory rates than the high-dose gemcitabine treatment; while, the survival period of treated mice with a combination of Ad-VT and low-dose gemcitabine was significantly prolonged. For the H&E staining, there was no obvious liver or kidney injury in the low dose gemcitabine or combination group. These results suggest that Ad-VT combined with low dose gemcitabine have no obvious injury to the liver and kidney of the mice, thus achieving the goal of reducing toxicity indirectly.

To model oncolytic adenovirus therapy, researchers typically use human tumor xenografts that support viral replication in immunocompromised mice. In this study, human lung cancer cells were used to establish xenograft tumor model in nude mice, and the growth trend of tumor in different treatment groups was observed intuitively, indicating that the combination of oncolytic adenovirus and chemotherapy drugs is a promising method. However, the findings of this study have to be seen in light of some limitations. Preclinical safety studies are hampered by a lack of permitted non-human hosts. Some studies have shown that limiting the productive replication of Ad5 in rodent and rabbit cells appears to be primarily a step after entry (40), and some murine cancer cells are generally unable to produce infectious viral progeny from human adenoviruses (41). Our study on the toxicity of Ad-VT combined with gemcitabine is limited by the lack of mouse model system, especially for nude mice, so it is limited to evaluate the toxicity of virus combined therapy. To systematically evaluate the benefits and side effects of combination therapy, we will establish more animal models for further validation in future studies.

## Data Availability Statement

The datasets generated for this study are available on request to the corresponding author.

## Ethics Statement

The animal experimental protocols were approved by the Institutional Animal Care and Use Committee of the Chinese Academy of Military Medical Science, Changchun, China (10ZDGG007).

## Author Contributions

XLiu, XLi, GSh, and NJ conceived and designed the experiments. XLiu, YL, YZ, WL, JW, YC, CS, ZL, GSo, and CL performed the experiments. XLiu, ZY, XLi, GSh, and NJ analyzed the data. XLiu, YL, YZ, and WL contributed reagents, materials, and analysis tools. XLiu and ZY wrote the manuscript. All authors read and approved the final manuscript.

### Conflict of Interest

The authors declare that the research was conducted in the absence of any commercial or financial relationships that could be construed as a potential conflict of interest.
